# The National Library of Medicine Global Health Events web archive, coronavirus disease (COVID-19) pandemic collecting

**DOI:** 10.5195/jmla.2020.1090

**Published:** 2020-10-01

**Authors:** Susan L. Speaker, Christie Moffatt

**Affiliations:** 1 speakes1@nih.gov, Historian for the Digital Manuscripts Program, History of Medicine Division, National Library of Medicine, Bethesda, MD; 2 christie.moffatt@nih.gov, Manager of the Digital Manuscripts Program, History of Medicine Division, and Chair, Web Collecting and Archiving Working Group, National Library of Medicine, Bethesda, MD

## Abstract

Since January 30, 2020, when the World Health Organization declared the SARS CoV-2 disease (COVID-19) to be a public health emergency of international concern, the National Library of Medicine's (NLM's) Web Collecting and Archiving Working Group has been collecting a broad range of web-based content about the emerging pandemic for preservation in an Internet archive. Like NLM's other Global Health Events web collections, this content will have enduring value as a multifaceted historical record for future study and understanding of this event. This article describes the scope of the COVID-19 project; some of the content captured from websites, blogs, and social media; collecting criteria and methods; and related COVID-19 collecting efforts by other groups. The growing collection—2,500 items as of June 30, 2020—chronicles the many facets of the pandemic: epidemiology; vaccine and drug research; disease control measures and resistance to them; effects of the pandemic on health care institutions and workers, education, commerce, and many aspects of social life; effects for especially vulnerable groups; role of health disparities in infection and mortality; and recognition of racism as a public health emergency.

## INTRODUCTION

**Figure 1:**
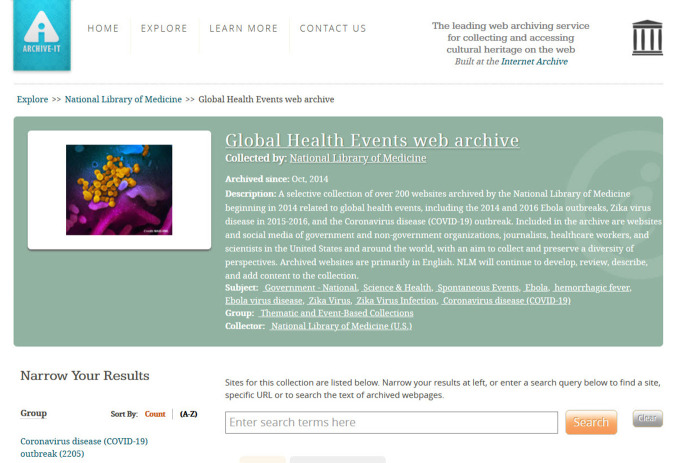
National Library of Medicine Global Health Events web archive

On January 30, 2020, as the World Health Organization (WHO) declared the novel severe acute respiratory syndrome coronavirus 2 (SARS-CoV-2) a public health emergency of international concern [1], the National Library of Medicine (NLM) began collecting web and social media documenting the emerging health crisis as part of its ongoing Global Health Events web archive collection (Figure 1) [2]. Web collecting is supported by the *Collection Development Guidelines of the National Library of Medicine*, which regard websites, blogs, social media, and other web content as increasingly important sources for documenting the scholarly biomedical record and illustrating diverse cultural perspectives in health and medicine [3]. Collecting and archiving content related to the coronavirus disease (COVID-19) pandemic follows earlier collecting efforts on the 2014 Ebola outbreak [4], the 2016 Zika virus outbreak [5], HIV/AIDS [6], and the ongoing opioid epidemic [7]. This article describes the authors' collecting efforts relating to the coronavirus disease through June 2020.

The Global Health Events web archive is available at <https://archive-it.org/collections/4887/>.

As with NLM's earlier web collecting projects, its staff is collecting a broad range of born-digital web and social media resources to document the diversity of roles, experiences, and perspectives on the pandemic. Originally intended to share news and information about the pandemic as it happens, this content will have enduring value as a multifaceted historical record for future study and understanding of this event and its myriad effects on our lives. As we know, web and social media documents change frequently over time and can disappear completely without notice; therefore, NLM, along with many other libraries and archives, is working now, in real time, to identify, collect, and archive content to preserve it for future research.

## APPRAISAL AND SELECTION

To build this collection following NLM's collection development guidelines, NLM's Web Collecting and Archiving Working Group—a team consisting of archivists, librarians, and a historian—is identifying web content around major public health events—including “disaster management,” “infectious diseases,” “health communication,” “history of medicine,” and “popular literature”—in consultation with NLM's disaster health librarian and infectious disease subject matter expert. The working group is also seeking recommendations from across NLM and the Office of National Institutes of Health (NIH) History [8], and welcomes external suggestions through strategic communications, including a post shared in the History of Medicine Division's *Circulating Now* blog [9]. Through these efforts, the working group aims to document a variety of aspects of the pandemic, including research, facilities, support for and contributions of the health workforce, health disparities, social dimensions, personal narratives, ethical considerations, caregiving at home, the role and protection of nonmedical essential workers, efforts to count cases and track the disease, and rumors and misinformation.

Collecting material about this pandemic has been much more challenging than our previous NLM Global Health Events projects. More than observing from the sidelines, we are experiencing the event ourselves, as individuals—caring for loved ones, schooling children at home, and doing our part to slow the spread—and as staff in an institution actively involved in the response [10]. The coronavirus pandemic is a rapidly evolving and enormously disruptive worldwide event. Its effects extend far beyond the medical and public health sphere into every aspect of our lives: economic, political, social, cultural, and spiritual. Because the virus is new and can be fatal, all face a steep learning curve not only in clinics and research labs, but also in our everyday activities, which must be limited and reconfigured to constrain the spread of the virus. The pandemic is revealing both the strengths and the shortcomings of the world's various health care systems and of our scientific and public institutions. It has exposed longstanding racial and economic disparities and has likewise sharpened social and political fault lines.

**Figure 2:**
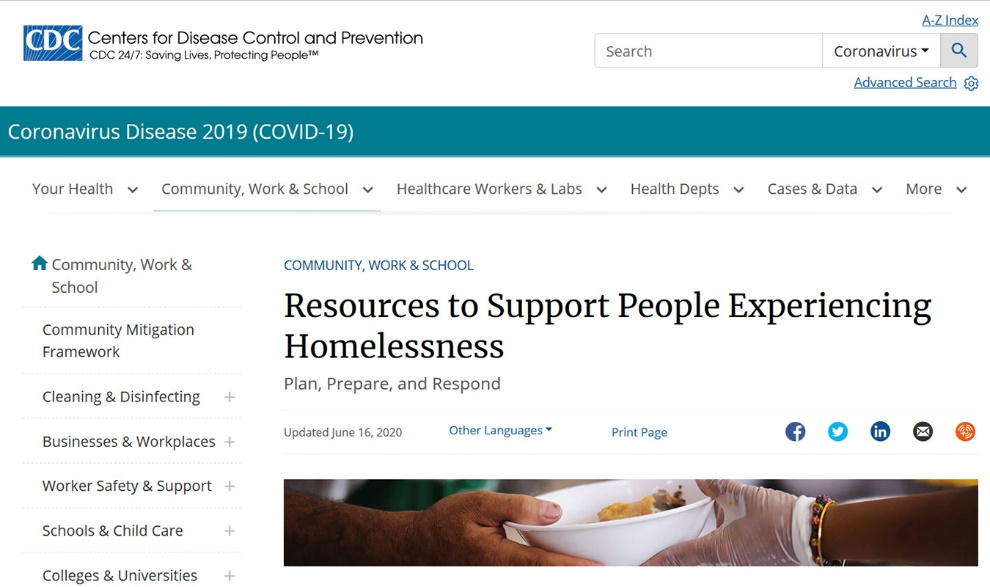
Screen capture of US Centers for Disease Control and Prevention: Resources to Support People Experiencing Homelessness

The NLM Web Collecting and Archiving Working Group began by identifying the coronavirus websites of international, federal, state, and select local government and nongovernment organizations involved in the response, including the World Health Organization, US Centers for Disease Control and Prevention, NIH, all fifty-five states and territories, and Native American tribal governments. These websites document current status and updates on the spread of the coronavirus, health and safety information, and news of technological innovations, data sharing, and development of treatments and vaccines. Content includes resources for specific groups or individuals such as those who are deaf or hard of hearing, vulnerable populations such as those with underlying health conditions, or people experiencing homelessness, incarceration, or addiction, such as the US Centers for Disease Control and Prevention Resources to Support People Experiencing Homelessness (Figure 2).

The archive of captures of the US Centers for Disease Control and Prevention Resources to Support People Experiencing Homelessness is available at <https://wayback.archive-it.org/4887/*/https://www.cdc.gov/coronavirus/2019-ncov/community/homeless-shelters/index.html>.

Along with government and health organizations, many other groups and individuals are contributing to the online coronavirus pandemic chronicle, providing myriad stories and perspectives. Our collection currently includes stories from nurses, physicians, and other health personnel at hospitals overwhelmed with COVID-19 cases [11, 12]; patients who survived the disease [13]; and funeral home directors overwhelmed by high numbers of COVID-19 deaths. As the pandemic has evolved, the impact of longstanding health and economic disparities has become starkly clear, with African American, Hispanic, and Native American communities suffering disproportionate levels of COVID-19 infection and mortality [14]. This outcome has coincided with widespread protests addressing police brutality, which have yielded renewed efforts to recognize racism as a public health emergency and, more broadly, to appreciate social determinants of health in defining health disparities.

As it has dominated hospital facilities and required social distancing and quarantine, the pandemic has also done collateral damage to the “normal” health care landscape [15]. Reflecting this damage are the many stories and studies of what might happen when people delay treatment for heart attacks or strokes, for example, due to fears of COVID-19 in health care facilities, or when they delay routine vaccinations, surgery, and other care [16]. Mental health professionals are tracking increases in depression and anxiety and working to stay in touch with vulnerable patients. Also, those who depend on addiction treatment groups such as Alcoholics Anonymous have struggled to maintain support networks when it is not possible to meet in person.

Prevention strategies form another topical area in the coronavirus collecting effort. In the first months of the pandemic, we found many videos and other visuals about washing hands, sanitizing surfaces, and taking other preventive actions. Once it became clear that wearing face masks decreased the spread of virus-containing droplets, information about masks was soon all over the Internet: how to make them, how to wear them, and which ones are best [17–19]. Critical shortages of personal protective equipment (PPE) for health care workers, sanitizing supplies, and equipment such as ventilators hampered early efforts to care for COVID-19 patients and avoid spreading the disease. At the time of this writing, PPE shortages still constrain COVID-19 prevention and treatment in some parts of the United States [20, 21]. These obstacles are another part of the pandemic story, including the ways that people have pitched in and helped to alleviate some of the shortages with innovations in manufacturing, repurposing, and alternative supply networks. Innovation, both practical and psychological, has also been required as people adapt to staying (and working) at home full time.

Resistance to and backlash against public health measures such as staying home and wearing masks in public places began within a month of the first stay-at-home orders in many states. The web content on this topic illustrates the essential, and in many cases historical, tensions present in large public health emergencies: between public health workers trying to inform the public, contain a dangerous disease, and save lives, and members of the public who see emergency restrictions (face coverings, testing, limiting public activities, and closures of public and private spaces) as infringements of individual freedoms and threats to their livelihoods. Related to these stories, we have also collected reports of preemptive resistance from vaccine opponents who fear that when a coronavirus vaccine is developed, the government will make it mandatory.

Just as web and social media furnish a rich fund of information about the pandemic's progress and all the varied responses to it, they also provide a wealth of misinformation and conspiracy theories. Because SARS-CoV-2 is new, there is a lot of room for rumor and misunderstanding about where it came from, how dangerous it is, who is most vulnerable, and what might prevent or cure it [22].

## SCOPING, DATA CAPTURE, AND QUALITY CONTROL

We followed decisions about what content to capture with analysis and recommendations of how much of a site needs to be captured, how often, and for how long. The working group chooses to capture some materials only once, like a single blog post or news article; for others, the group decides to collect multiple copies over time (weekly, monthly, etc.) to document the evolution of content throughout the pandemic. We can see very specific changes such as the official naming of the virus in early February [23]; the WHO declaration on March 11, 2020, that COVID-19 had reached pandemic levels [24]; and broader changes in content and emphasis as organizations and individuals learn more about the coronavirus, how it spreads, and the varying impact on populations.

**Figure 3:**
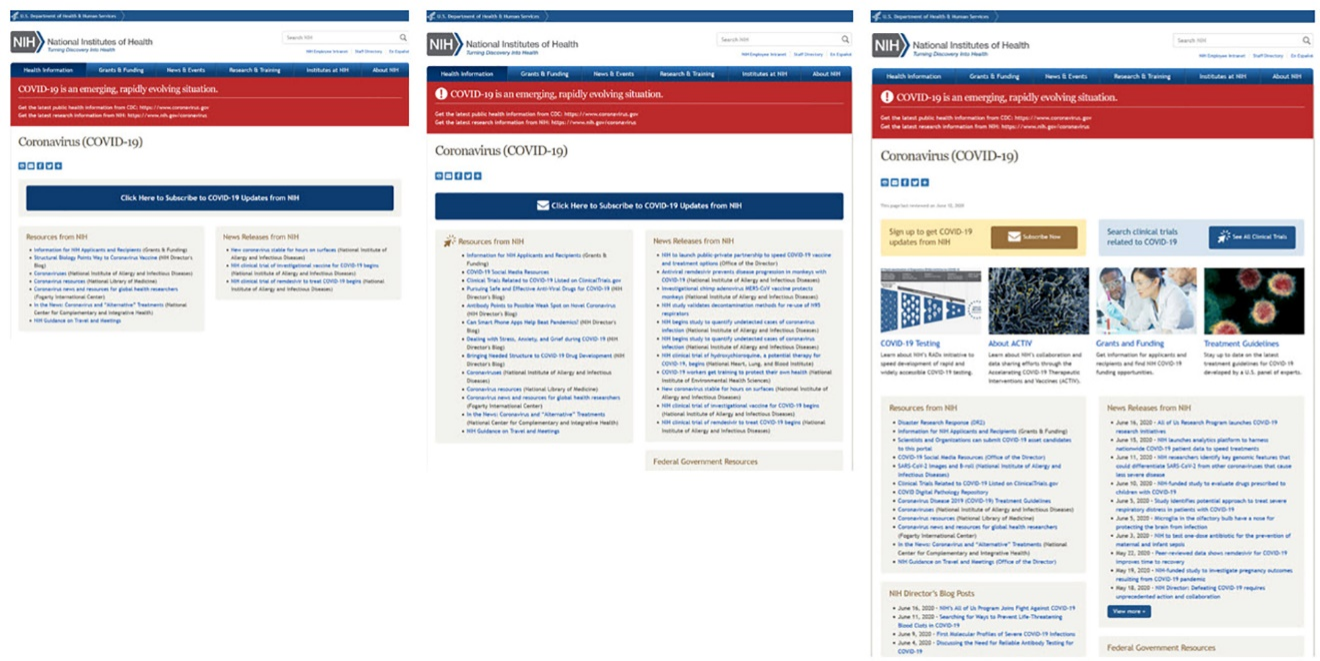
Screen captures of the National Institutes of Health (NIH) Coronavirus (COVID-19) website, March–June 2020

Monthly captures of the evolving NIH Coronavirus (COVID-19) website reveal a growing number of resources from March to June 2020, as NIH added news releases about research initiatives and clinical trials, NIH director blog posts, and information on NIH's Rapid Acceleration of Diagnostics (RADx) initiative and Accelerating COVID-19 Therapeutic Interventions and Vaccines (ACTIV) collaboration and data sharing efforts (Figure 3).

The archive of captures of the National Institutes of Health (NIH) coronavirus (COVID-19) website is available at <https://wayback.archive-it.org/4887/*/https://www.nih.gov/health-information/coronavirus>.

The working group is mainly using the Internet Archive's “Archive-It” Service, which is a tool that allows libraries and archives to collect web content, preserve it in an archival format (WARC file), and make the saved content available to current and future researchers. While the Internet Archive is capturing a significant amount of content broadly across the web, NLM and many other libraries can use Archive-It to collect more deeply in specific areas of interest central to their mission. The working group is also experimenting with another tool called Conifer (formerly, Webrecorder) to collect more interactive and technically challenging content [25].

Following capture, the working group reviews collected content for quality, comparing the archived copy with the live version; adds metadata at the item and collection level to support discovery; notifies creators of the intention to include it in our collection if content is personal in nature; and provides access to the content through Archive-It's public user interface.

## RELATED COLLECTING EFFORTS

While NLM's collection is mainly focused on the US experience, the working group is also contributing to a broader international collaborative effort to build a SARS CoV-2 outbreak web archive collection led by the International Internet Preservation Consortium [26, 27], with contributions from national and academic libraries around the world in over forty different languages. NLM is also engaged with other cultural heritage organizations that are archiving the history of COVID-19—in particular, the Society of American Archivists Web Archiving Roundtable, Archive-It community, National Digital Stewardship Alliance, and colleagues in the medical museum community—and is contributing to and following the growing list of institutions collecting COVID-19-related content that is maintained by the Documenting the Now project [28].

## CONCLUSION

At the time of this writing, the NLM Web Collecting and Archiving Working Group's collecting effort includes nearly 2,500 sources, and COVID-19 is still an emerging, rapidly evolving situation. Resources, information, and documentation of experiences of the coronavirus—the primary resources of our time—are changing constantly, and new content continues to emerge. The scope of our collecting is evolving, too, as we learn more about the virus and its spread, who it affects most and why, and the long-term impacts. NLM's collection will, of course, only be a small sample of the tremendous amount of COVID-19-related content shared via the web and will complement the many other documentation efforts by libraries, archives, museums, and communities across the country and around the world to preserve web and non-web-based (e.g., diaries, journals) materials about the pandemic.

We also acknowledge that some perspectives will not be documented or shared for a wide range of reasons, including technical barriers, opportunity, or personal choice of the creators. The working group will continue to identify, collect, and archive content documenting the COVID-19 outbreak throughout the duration of the pandemic and the development and deployment of drug treatments and vaccines. We welcome recommendations (email, nlmwebcollecting@nlm.nih.gov) for content to include and new aspects to consider. We value the diversity of perspectives on what is valuable to document on this still-unfolding event so that our collaborative work today can yield the best possible future historical record for research and understanding.
